# Pulmonary epidermoid carcinoma in a patient with acromegaly: a rare entity

**Published:** 2012-06-10

**Authors:** Siham El Aziz, Asma Chadli, Atika Obbiba, Hassan El Ghomari, Ahmed Farouqi

**Affiliations:** 1 Department of endocrinology, diabetes and metabolism, Ibn Rochd Universitary hospital, Casablanca, Morocco

**Keywords:** Acromegaly, carcinoma, pulmonary neoplasia, pituitary adenoma, epidermoid carcinoma

## Abstract

A 56-years-old woman was referred to our unit for partially treated acromegaly. She had a high level of insulin growth factor. She did not complain of any pulmonary symptoms and was a non-smoker. Physical examination revealed clinical features of acromegaly. She had a 13 mm pituitary adenoma and was proposed for surgical intervention. Her chest X-ray showed a right paracardiac tumor. Computed tomography scan revealed a large right-sided fowler tumor. Pituitary surgery was cancelled and lobectomy after biopsy with lymph nodes excision was performed through thoracotomy. Histological study of the tumor revealed a medium differentiated epidermoid carcinoma with positive lymph nodes and extension to pleura. She was referred to chemotherapy protocol. Association between carcinoma and acromegaly has previously been reported. Most common tumors are colorectal and thyroid neoplasia. As we see in this case report, we need to consider other carcinomas in acromegalic patients like pulmonary carcinoma, despite their rarity in women.

## Introduction

Acromegaly is a chronic disease caused usually by a pituitary adenoma secreting excess growth hormone (GH). Untreated acromegaly is associated with a high morbidity and two -fold mortality risk [1, 2]. Acromegalic patients have most frequently respiratory and vascular diseases [3]. More recently, many studies have shown an increase risk of neoplasias [4, 5] in acromegalic patients [6, 7], correlated to increase level of Insulin-like Growth Factor (IGF) and elevated proliferative activity [8–10]. The most common tumors are colorectal neoplasias, breast and thyroid cancer [11–13]. Screening of tumors must be done before and after tranpshenoidal surgery, and long term follow up is needed in partially treated acromegalic patients. Pulmonary carcinomas are much rare than other carcinoma in non-smoking acromegalic patients. We report a case of epidermoid carcinoma in an acromegalic woman and discuss this rare association.

## Patient and observation

**Figure 1:**
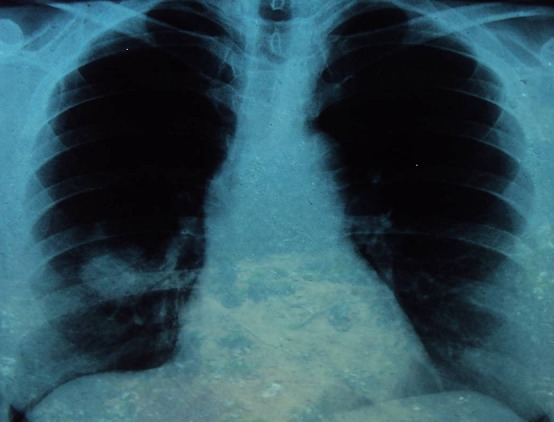
Right sided paracardiac opacity at chest X-ray without pleural effusion

**Figure 2:**
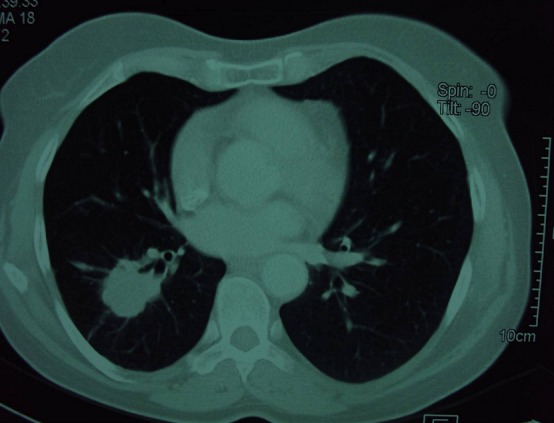
Right medium lobe large tumor at the thorax tomography scan, with speculated margin, in contact with the big lung sulcus and elargment of right inferior lobe

**Figure 3:**
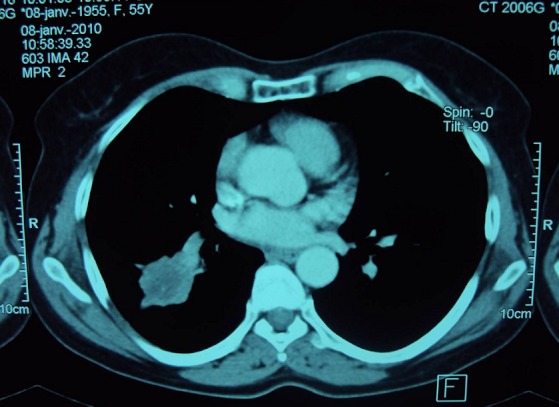
CT scan right ovalar 45x35 mm necrosed tumor with irregular margin, with no mediastinal lymph nodes, and no left parenchymatous anomaly and no pleural reaction

We report the case of a 56-years-old non-smoking female patient diagnosed with acromegaly seven years ago and treated by transphenoidal surgery and radiotherapy. She was referred to our unit for persistent acromegaly. She had evident signs of acromegaly on clinical examination. Laboratory investigation demonstrated increase IGF1 at 429 ng/ml and pituitary 13 mm macro-adenoma. She didn't have metabolic complications. Our decision was surgery, because of non availability of medical treatment. She was referred to the neurosurgeon and proposed a trans-sphenoidal adenoma removal. A routine chest-X-ray, done in the pre-operative workout, revealed a right sided paracardiac opacity (Figure 1). A thorax computed tomography scan (CT) showed a large tumor of the right medium lobe, in contact with the big lung sulcus and an enlargement of the right inferior lobe (Figure 2 and Figure 3). Abdominal CT was undertaken before surgery to exclude other localisations. Biopsy through thoracotomy revealed a malignant tumor. Resection of the right inferior pulmonary lobe with mediastinal lymph nodes excision were realized. Histological examination demonstrated a 5 cm epidermoid carcinoma poorly differentiated with extension to pleura and with metastatic lymphonodes. Pituitary surgery was cancelled; the patient was referred to an oncological center for chemotherapy protocol.

## Discussion

Acromegaly is a rare disease with a prevalence of 40 cases/1 million population and 3 new cases/1 million population per year due to excess growth hormone (GH) secreted usually by a pituitary adenoma [14, 15]. Majority of clinical and metabolic complications of acromegaly are caused by increase levels of GH witch induce high insulin like growth factor 1 (IGF1) [16]. Beside morbidity of acromegaly, mortality seems to be increased in this disease. Several studies concerning long term follow-up of acromegalic patients demonstrated a link between acromegaly and cancer since IGF1 increase proliferative activity of cells [17, 18]. Most common tumors are colorectal [19], thyroid carcinoma [20], breast and prostate tumors. It is recommended for acromegalic patients to undergo screening colonoscopy and thyroid ultrasonography [11]. Great attention had been provided to colorectal cancer since a high level of IGF1 has been correlated to increase risk of colorectal cancer [9, 21–23]. The American Cancer Society defined acromegalic patients just above the average risk of colorectal cancer [24] and recommend that colonoscopsy should be prescribed after the age of 50 [25].

Lung cancer is the most frequent cancer inducing deaths in women and men according to the American Cancer Society [24]. Lung cancer in acromegalic patients seems to be rare; and it's seems like there is not an increase prevalence of non-small cell cancer in acromegalic patients compared to normal population according to several studies [5, 17, 26] even if high level of IGF1 value increase proliferation of lung cells [27, 28]. Table 1 show the prevalence of most frequent cancer in acromegalic patients according to different studies. As we can see, neoplasias are frequent in acromegalic patients with a prevalence of about 10% according to these large scale epidemiological studies (Table 1). Lung carcinoma seems extremely rare in patients with acromegaly. Our female patient was a non-smoking patient, she didn't complain of chest pain, cough, trouble breathing or weight. The major risk factor for this patient seems to be the chronic exposure to high level of IGF1 resulting from partially treated acromegaly. Epidermoid carcinoma is a non-small lung cancer with small chance of recovery and poor prognosis when the cancer is in an advanced stage or in poorly-differentiated tumors, like in our patient. Since lung cancer may not be associated with any symptoms, a routine chest X-ray should be done for screening in acromegalic patient, including in treated patient with persistent high level of IGF1 [28].

**Table 1:** Cancer prevalence in acromegaly according to several studies

Variables	Baris [17]	Popovic [6]	Baldys-Waligorska [7]	Barzilay [18]	Orme [5]
Year of study	2001	1997	2010	1991	1998
Number of patients with acromegaly	1634	220	101	87	1362
Medium age at diagnosis (years)	50,4	49,5 ± 0,9	51,8 ± 15,4	37	-
Mean follow-up (years)	10,3	4,5 ± 0,4	9,4 ± 6,5	13	-
**Number of cancer**					
n	177	23	12	7	79
%	10,8	10,4	11,8	8	5,8
**Malignancy localisation (n)**					
Colorectal	34	2	2	1	16
Thyroid	3	3	3	2	1
Breast	20	4	1	1	14
Cervix	3	4	3	0	-
Endometer	4	0	0	0	-
Bladder	3	1	0	0	-
Stomach	6	0	1	0	-
Kidney	12	1	0	0	-
Lung	14	0	1	0	-
Ovary	2	1	0	1	6
Hematopoietic	23	3	0	1	-
Skin	13	2	0	1	-
Prostate	13	0	1	0	-
Brain	9	0	0	0	-
Bone	2	0	0	0	-
Other digestive	16	2	0	0	-

## Conclusion

Active disease may precede for several years the diagnosis of acromegaly because of indolence. Highl levels of IGF1 contribute to progression of malignant tumors. In acromegalic patients, screening is fundamental, and chest X-ray with CT may be necessary. During follow-up, more attention should be given in partially treated acromegalic patients because of persistent increase of IGF1 levels. Screening of neoplasias should be done in these high risk patients.
